# Predicting Slow Walking Speed From a Pooled Cohort Analysis: Sarcopenia Definitions, Agreement, and Prevalence in Australia and New Zealand

**DOI:** 10.1093/gerona/glad165

**Published:** 2023-07-10

**Authors:** Jesse Zanker, David Scott, Cassandra Szoeke, Sara Vogrin, Sheena Patel, Terri Blackwell, Stefanie Bird, Ben Kirk, Jacqueline Center, Dima A Alajlouni, Tiffany Gill, Graeme Jones, Julie A Pasco, Debra L Waters, Peggy M Cawthon, Gustavo Duque

**Affiliations:** Australian Institute for Musculoskeletal Science (AIMSS), The University of Melbourne and Western Health, St. Albans, Victoria, Australia; Department of Medicine, Western Health, The University of Melbourne, St. Albans, Victoria, Australia; Institute for Physical Activity and Nutrition, Deakin University, Burwood, Victoria, Australia; Department of Medicine, School of Clinical Sciences at Monash Health, Monash University, Clayton, Victoria, Australia; Department of Medicine, Royal Melbourne Hospital, The University of Melbourne, Parkville, Victoria, Australia; Australian Institute for Musculoskeletal Science (AIMSS), The University of Melbourne and Western Health, St. Albans, Victoria, Australia; Department of Medicine, Western Health, The University of Melbourne, St. Albans, Victoria, Australia; Research Institute, California Pacific Medical Center, San Francisco, California, USA; Research Institute, California Pacific Medical Center, San Francisco, California, USA; Australian Institute for Musculoskeletal Science (AIMSS), The University of Melbourne and Western Health, St. Albans, Victoria, Australia; Institute for Physical Activity and Nutrition, Deakin University, Burwood, Victoria, Australia; Australian Institute for Musculoskeletal Science (AIMSS), The University of Melbourne and Western Health, St. Albans, Victoria, Australia; Department of Medicine, Western Health, The University of Melbourne, St. Albans, Victoria, Australia; Skeletal Diseases Program, Garvan Institute of Medical Research, Sydney, New South Wales, Australia; Faculty of Medicine and Health, University of New South Wales, Sydney, New South Wales, Australia; Skeletal Diseases Program, Garvan Institute of Medical Research, Sydney, New South Wales, Australia; Faculty of Medicine and Health, University of New South Wales, Sydney, New South Wales, Australia; Adelaide Medical School, University of Adelaide, Adelaide, South Australia, Australia; Menzies Institute for Medical Research, University of Tasmania, Hobart, Tasmania, Australia; Department of Medicine, Western Health, The University of Melbourne, St. Albans, Victoria, Australia; IMPACT-Institute for Mental and Physical Health and Clinical Translation, Barwon HealthDeakin University, Geelong, Victoria, Australia; Department of Medicine, School of Physical Education, Sport and Exercise Sciences, University of Otago, Dunedin, New Zealand; Department of Internal Medicine/Geriatrics, University of New Mexico, Albuquerque, New Mexico, USA; Research Institute, California Pacific Medical Center, San Francisco, California, USA; Department of Epidemiology and Biostatistics, University of California, San Francisco, California, USA; Australian Institute for Musculoskeletal Science (AIMSS), The University of Melbourne and Western Health, St. Albans, Victoria, Australia; Department of Medicine, Research Institute of the McGill University Health Centre, McGill University, Montreal, Quebec, Canada

**Keywords:** Grip strength, Muscle strength, Physical performance, Sarcopenia, Walking speed

## Abstract

**Background:**

Recent operational definitions of sarcopenia have not been replicated and compared in Australia and New Zealand (ANZ) populations. We aimed to identify sarcopenia measures that discriminate ANZ adults with slow walking speed (<0.8 m/s) and determine the agreement between the Sarcopenia Definitions and Outcomes Consortium (SDOC) and revised European Working Group for Sarcopenia in Older People (EWGSOP2) operational definitions of sarcopenia.

**Methods:**

Eight studies comprising 8 100 ANZ community-dwelling adults (mean age ± standard deviation, 62.0 ± 14.4 years) with walking speed, grip strength (GR), and lean mass data were combined. Replicating the SDOC methodology, 15 candidate variables were included in sex-stratified classification and regression tree models and receiver operating characteristic curves on a pooled cohort with complete data to identify variables and cut points discriminating slow walking speed (<0.8 m/s). Agreement and prevalence estimates were compared using Cohen’s Kappa (CK).

**Results:**

Receiver operating characteristic curves identified GR as the strongest variable for discriminating slow from normal walking speed in women (GR <20.50 kg, area under curve [AUC] = 0.68) and men (GR <31.05 kg, AUC = 0.64). Near-perfect agreement was found between the derived ANZ cut points and SDOC cut points (CK 0.8–1.0). Sarcopenia prevalence ranged from 1.5% (EWGSOP2) to 37.2% (SDOC) in women and 1.0% (EWGSOP2) to 9.1% (SDOC) in men, with no agreement (CK <0.2) between EWGSOP2 and SDOC.

**Conclusions:**

Grip strength is the primary discriminating characteristic for slow walking speed in ANZ women and men, consistent with findings from the SDOC. Sarcopenia Definitions and Outcomes Consortium and EWGSOP2 definitions showed no agreement suggesting these proposed definitions measure different characteristics and identify people with sarcopenia differently.

Since the coining of the term “sarcopenia” by Rosenberg in 1989 (1), significant efforts have been made to define and operationalize the phenotype commonly understood as low muscle mass and/or strength or physical function (2). Underpinning these efforts is the goal to optimize muscle health in older adults through targeted intervention. The lack of a global consensus definition of sarcopenia, however, has hampered research and clinical progress (3). Further, despite the willingness of older adults to actively engage in musculoskeletal health interventions (4,5), knowledge of sarcopenia among clinicians and the public remains poor (4,6).

The numerous operational definitions of sarcopenia presented over the decades have been either consensus-driven (7–15) or data-driven (2,16,17), and target general (2,8,18) or specific (9,10,15) populations of older, community-dwelling adults. In 2019, the European Working Group for Sarcopenia in Older People (EWGSOP) revised their original operational definition (EWGSOP1) through a consensus process that identified preferred measures and cut points for muscle strength, physical performance, and muscle quality and quantity (EWGSOP2) (8). In 2020, the Sarcopenia Definitions and Outcomes Consortium (SDOC) presented a data-driven definition of sarcopenia comprising cut points for low muscle strength via handheld dynamometry (and various adjustments) that predict low walking speed (<0.8 m/s). In 2022, the Australian and New Zealand Society for Sarcopenia and Frailty Research (ANZSSFR) Task Force published guidelines established by consumer- and topic-expert Delphi consensus in which the EWGSOP2 definition was adopted for use in the region (5,13). The parameters and cut points for both EWGSOP2 and SDOC are presented in Supplementary Table 1.

The estimated prevalence of sarcopenia depends on the population studied and the definition applied. Sarcopenia prevalence increases with age and is higher among those who are hospitalized, have multimorbidity or frailty, or live in residential aged care (19–23). Data from the UK Biobank comprising 316 980 community-dwelling adults aged 50 years and older showed that the prevalence of sarcopenia determined by the EWGSOP2 definition was 0.4% and around 20 times more common in women in the cohort (24). In a subset of 20 400 adults from the Biobank (9 572 women, 10 828 men) mean age of 67.8 years with telomere data, the prevalence of sarcopenia using EWGSOP2 was 1% and 2% using SDOC (24). In addition to poor internal agreement and low agreement between EWGSOP1 and EWGSOP2 sarcopenia definitions, single cohort studies using the EWGSOP2 definition have found a low prevalence and wide estimate (3%–26%) of sarcopenia in older Australians attending a Falls and Fractures Clinic (25) depending on the measures used for low muscle strength and physical performance. This finding contrasts the high prevalence of sarcopenia in the same population when applying the SDOC definition (45.5%), which requires low handgrip strength and walking speed (26).

Given these recent developments and ongoing uncertainty, there is a need to establish the most appropriate sarcopenia characteristics and cut points for use in Australia and New Zealand (ANZ). Indeed, the SDOC emphasized the importance of evaluating their definition in diverse populations (18), and the prevalence of sarcopenia in ANZ according to established definitions using large, pooled cohorts has not been previously investigated. To answer these questions, we first replicated the analytic methods applied by the SDOC in 2 Australian cohorts to establish the variables and cut points that best predict slow walking speed (<0.8 m/s). Second, we applied these cut points, and the variables and cut points presented by EWGSOP2, to 4 pooled cohorts from 8 ANZ studies to determine sarcopenia prevalence and definition agreement in community-dwelling adults. We hypothesized that the variables and cut points selected by CART models that best discriminate slow walking speed would replicate those generated by SDOC models. Second, we hypothesized that the prevalence of sarcopenia by cut points determined by CART models in this study would be higher than prevalence estimates by EWGSOP2 and would show poor agreement between definitions. Consistent with the SDOC approach, we followed the guidelines presented by the “Transparent Reporting of a Multivariable Prediction Model for Individual Prognosis or Diagnosis” (TRIPOD) initiative (27).

## Method

### Study Population

Data from 7 longitudinal studies and 1 cross-sectional study of Australian and New Zealand adults were pooled into 4 cohorts (Supplementary Table 2). All adults were included in the analysis and were not restricted to age greater than 65 years, in contrast to the SDOC analysis. This broadening of age inclusion reflects recent consensus guidelines from the ANZSSFR Task Force (5,13), recommending that persons under 65 should be assessed for sarcopenia if living with comorbidities that increase sarcopenia risk, or are of Aboriginal, Torres Strait Islander, or Māori heritage. All included studies comprised participants with median age greater than 55 years. All 4 cohorts comprised studies including dual-energy X-ray absorptiometry (DXA)-derived body composition. Cohort 1 (*performance and strength cohort*, *n* = 1 858) comprised a cross-sectional Falls and Fracture Clinic (26) population from Melbourne, Australia, and the longitudinal Geelong Osteoporosis Study (GOS) (20) that included grip strength (GR) and walking speed data and were used in the CART model (2). Cohort 2 (*performance cohort*, *n* = 1 531) comprised data from the Dubbo Osteoporosis Epidemiology 2 (28) and Vital D studies (29), which included walking speed. Cohort 3 (*strength cohort*, *n* = 4 528) comprised data from the North West Adelaide Health Study (30), the Tasmanian Older Adult Cohort (TASOAC) (31), and the Women’s Healthy Ageing Project (WHAP) (32) studies, which included GR. Cohort 4 comprised a single study from New Zealand, Age Concern Otago (OTAGO) (33) that included the Timed Up and Go (TUG) test (*TUG cohort*, *n* = 183). Data from the visit with most complete body composition, strength, and physical performance variables captured in each study were used.

### Grip Strength

GR was assessed with handheld dynamometry. Protocols for each cohort are detailed in Supplementary Table 2. The maximum value (kg) from either hand was used in the analysis.

### Walking Speed

Walking speed was assessed as the participant’s usual pace over 6 m measured in m/s, without use of walking and mobility aids. Low walking speed was defined as <0.8 m/s (as was defined in the SDOC analysis); a cut point that has strong associations with adverse health outcomes in adults (34). Different cut points of walking speed (<0.6 m/s, <1.0 m/s, and continuous variable) were not treated as outcomes in the model in contrast to the SDOC analysis, as <0.8 m/s was the cut point ultimately adopted by the SDOC.

### Body Size and Composition

Anthropometric measures (height [m], weight [kg], and body mass index [BMI, kg/m^2^]) were measured using standardized procedures in each cohort (Supplementary Table 2). As body surface area (BSA) was not an adjustment that altered prediction models in the SDOC analysis, it was not included as a candidate adjustment in this analysis. For body composition, only appendicular lean mass (ALM) calculated using DXA was available (body fat and separate arm and leg lean mass measures were not universally available).

Variability within and between DXA manufacturers on body composition estimation and impact on prediction models was examined in the SDOC analysis (2). Harmonization of DXA-derived body composition variables via adjustment to a common standard using normative data did not meaningfully affect predictive models in the SDOC analysis (2). Further, harmonization in this cohort was not possible due to how data were collected, thus original nonharmonized values were used in our analysis.

### Statistical Analysis

Cohorts were stratified by sex, recognizing established sex differences in body composition, muscle strength, and physical performance. As described above, cohorts were harmonized based on the presence of strength and physical performance variables into 1 of 4 cohorts. Each cohort was presented descriptively by median (interquartile range) for skewed variables and mean (standard deviation) for normally distributed variables. Receiver operating characteristic (ROC) were plotted on each variable for walking speed <0.8 m/s and the area under the curve (AUC) with 95% confidence interval (CI) and *p* value were calculated. The Youden Index (sensitivity + specificity − 1) was calculated as the optimal cut point for discriminating slow walking speed (<0.8 m/s) by weighing sensitivity and specificity equally (35); further details on the Youden Index are published elsewhere (2).

We replicated the CART model used by the SDOC in Cohort 1 (*performance and strength cohort*) as CART produces empirical, nonparametric, and interpretable results and is an established, agnostic analytic method applied in similar musculoskeletal studies (2,16,36). CART is a machine learning technique that allows candidate variables to “compete” for an outcome (ie, slow walking speed <0.8 m/s), and presents interactions and cut points between variables that best discriminate those with and without the outcome. Sex-stratified models with 15 candidate variables, including age, anthropometric measures, GR, and ALM (with standardization to body composition measures; Supplementary Table 3) were included in each CART model and were performed with 10-fold cross-validation. Variable importance, defined as the relative importance of a variable in predicting the outcome of slow walking speed within the predictive model, was produced by each model. Independent *t* tests were used to compare groups with and without slow walking speed (<0.8 m/s) and with and without low GR determined by the SDOC cut points.

The Youden Index cut points for GR derived in Cohort 1 (*performance and strength cohort*) were then applied in Cohort 3 (*strength cohort*) to determine agreement with SDOC cut points using proportionate agreement and the Cohen’s Kappa (CK) statistic with measures of uncertainty (95% CI and *p* value). Given that the analysis of Cohort 1 produced “near perfect agreement” with SDOC cut points, unadjusted GR SDOC cut points and cut points from the EWGSOP2 sarcopenia definition were applied to Cohorts 1 to 4. “Probable sarcopenia” as defined in EWGSOP2 and the ANZSSFR clinical guidelines was determined by presence of either slow walking speed or low GR (13). “Sarcopenia” was defined by SDOC as slow walking speed and low GR, and by EWGSOP2 as either slow walking speed or low GR and low ALM or ALM/height^2^ (2,8). Prevalence of “probable sarcopenia” and “sarcopenia” was determined for both SDOC and EWGSOP2 cut points and definitions using descriptive statistics, and agreement was calculated using proportionate agreement and the CK statistic.

Statistical analysis was performed using IBM SPSS (Version 28.0.1.1 (14)); harmonization and CART models were performed using RStudio (2022.02.1 Build 461).

## Results

**Figure 1.**
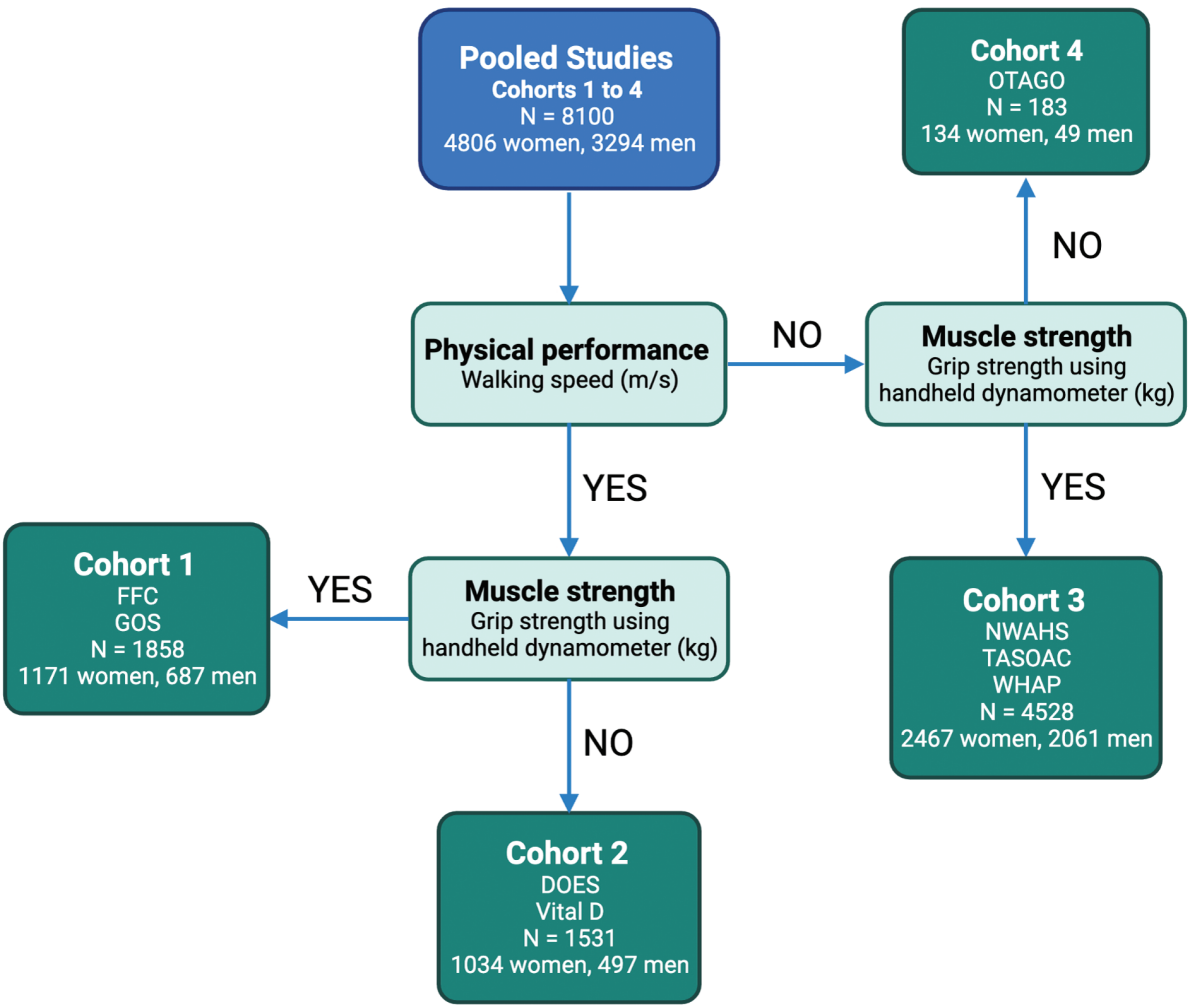
Participant flow and cohorts. FFC = Australian Institute for Musculoskeletal Science Falls and Fracture Clinic; GOS = Geelong Osteoporosis Study; DOES = Dubbo Osteoporosis Epidemiology Study; Vital D = Vital D Study; NWAHS = North West Adelaide Health Study; TASOAC = Tasmanian Older Adult Cohort; WHAP = Women’s Healthy Ageing Project; OTAGO = Age Concern Otago; Cohort 1 = performance and strength cohort; Cohort 2 = performance cohort; Cohort 3 = strength cohort; Cohort 4 = Timed Up and Go cohort.

The pooled studies included *n* = 4 806 women and *n* = 3 294 men, divided into 4 cohorts (Figure 1 and Table 1). In Cohort 2 (*performance cohort*), 314/995 (31.6%) women had slow walking speed (<0.8 m/s) and were significantly (*p* < .05) older, had higher BMI and lower ALM than women with normal walking speed (≥0.8 m/s); 149/461 (32.3%) men had slow walking speed and were significantly older, shorter, and had lower ALM (Supplementary Table 4) than men with normal walking speed. In Cohort 3 (*strength cohort*), 887/2 440 (36.4%) women had low unadjusted GR (<20 kg) and were significantly older, shorter, and had lower body weight and ALM than women with normal GR (≥20 kg); 881/2 045 (43.1%) men had low unadjusted GR (<35.5 kg) and were older, had lower BMI and ALM than men with normal GR (≥35.5 kg; Supplementary Table 4).

**Table 1.** Age, Anthropometry, Muscle Strength, Physical Performance, and Body Composition Characteristics of Women and Men in Four Pooled Cohorts

Variable	Cohort 1—Performance and Strength *N* = 1 858		Cohort 2—Performance *N* = 1 531		Cohort 3—Strength *N* = 4 528		Cohort 4—TUG *N* = 183	
	Women *N* = 1 171	Men *N* = 687	Women *N* = 1 034	Men *N* = 497	Women *N* = 2 467	Men *N* = 2 061	Women *N* = 134	Men *N* = 49
Age, years, median (IQR)	67.0 (49.9, 76.5)	64.6 (53.2, 73.9)	69.1 (66.0, 72.9)	68.8 (66.7, 71.8)	58.8 (48.0, 71.0)	58.0 (47.0, 68.0)	72.7 (68.0, 77.0)	74.0 (69.0, 79.0)
Setting	Community-dwelling^*^	Community-dwelling^*^	Community-dwelling	Community-dwelling	Community-dwelling	Community-dwelling	Community-dwelling	Community-dwelling
Weight, kg, *SD*	72.4 (16.0)	84.6 (14.8)	71.5 (13.9)	85.8 (14.0)	72.8 (15.0)	86.0 (15.4)	71.2 (14.0)	84.5 (12.4)
Height, m, *SD*	1.60 (0.07)	1.73 (0.08)	1.60 (0.06)	1.74 (0.06)	1.61 (0.06)	1.75 (0.07)	1.59 (0.06)	1.72 (0.07)
BMI, kg/m^2^, *SD*	28.4 (5.9)	27.9 (4.3)	28.0 (5.3)	28.5 (4.2)	28.1 (5.9)	28.2 (4.5)	28.2 (5.3)	28.4 (3.9)
Grip strength, kg, *SD*	23.9 (7.5)	38.2 (9.4)	—	—	22.4 (9.1)	35.9 (15.6)	—	—
Grip/weight, kg/kg, *SD*	0.34 (0.12)	0.47 (0.12)	—	—	0.32 (0.14)	0.43 (0.19)	—	—
Grip/BMI, kg/kg/m^2^, *SD*	0.88 (0.34)	1.40 (0.41)	—	—	0.83 (0.39)	1.30 (0.60)	—	—
Walking speed, m/s, *SD*	0.73 (0.29)	1.06 (0.35)	0.92 (0.23)	0.87 (0.16)	—	—	—	—
TUG, s, *SD*	11.5 (7.2)	15.2 (7.2)	8.3 (3.0)	7.3 (2.1)	9.7 (4.0)	—	8.2 (2.2)	7.2 (1.4)
ALM, kg, *SD*	17.31 (2.85)	25.34 (4.04)	15.91 (2.21)	23.89 (3.29)	16.75 (2.52)	25.17 (3.75)	15.49 (1.97)	23.84 (3.28)
ALM/height^2^, kg/m^2^, *SD*	6.77 (0.93)	8.42 (1.02)	6.20 (0.76)	8.04 (0.86)	6.53 (0.80)	8.39 (0.92)	6.62 (0.65)	8.01 (0.86)
ALM/BMI, kg/kg/m^2^, *SD*	0.63 (0.12)	0.92 (0.15)	0.60 (0.08)	0.88 (0.11)	0.60 (0.10)	0.91 (0.14)	0.56 (0.10)	0.85 (0.14)

*Notes*: Variables are given in mean ± standard deviation unless otherwise stated.

ALM = appendicular lean mass; BMI = body mass index; IQR = interquartile range; *SD* = standard deviation; TUG = Timed Up and Go test.

^*^Community dwelling in Falls and Fracture Clinic of Cohort 1 are “high risk” as attending a community outpatient clinic.

Table 2 presents ROC AUC, Youden Index, and optimal cut points (derived from Cohort 1, *performance and strength cohort*) for slow walking speed (<0.8 m/s) in women and men for each candidate variable, and SDOC cut points for GR variables. In women, AUCs for age, height, and GR variables ranged from 0.63 to 0.68 and are considered to have “satisfactory” diagnostic accuracy (*p* values <.001). AUCs for weight, BMI, and all ALM measures in women were 5%–10% lower, ranging from 0.51 to 0.59 and were considered to have “unsatisfactory” diagnostic accuracy. In men, AUCs were slightly lower (<5%) than in women. AUCs for height and GR variables (excepting GR/weight and GR/ALM) in men ranged from 0.61 to 0.64 and were considered to have satisfactory diagnostic accuracy (*p* values <.001). AUCs for age, weight, BMI, and all ALM variables in men ranged from 0.52 to 0.59 and had unsatisfactory diagnostic accuracy. The Youden Index for women (average = 0.20; range = 0.08–0.30) and men (average = 0.20, range = 0.10–0.28) was consistently low. The average Youden Index of GR variables (women, average = 0.27; men, average = 0.24) was consistently higher than the Youden Index for ALM variables (women, average = 0.12; men, average = 0.16). In both women and men, GR/body mass index (GRBMI) was the strongest discriminating variable for slow walking speed in both women (GRBMI <0.71 kg/kg/m^2^) and men (GRBMI <1.09 kg/kg/m^2^), and Youden Indices = 0.30 and 0.28, respectively.

**Table 2.** Cohort 1 (*Performance and Strength Cohort*) Area Under the Receiver Operating Characteristic Curve and the Youden Index for Walking Speed <0.8 m/s by Age, Body Anthropometry, Body Composition Measures, and Grip Strength in Men and Women

Variable	Women				Men			
	Area Under the Curve (95% CI), *p* Value	Youden Index	Optimal Cut Point	SDOC Cut Point	Area Under the Curve (95% CI), *p* Value	Youden Index	Optimal Cut Point	SDOC Cut Point
Age, y	0.67 (0.62, 0.72), <.001	0.28	76.50	—	0.60 (0.54, 0.66), .001	0.22	70.95	—
Weight, kg	0.55 (0.49, 0.61), .139	0.11	72.7	—	0.58 (0.52, 0.64), .009	0.17	68.05	—
Height, m	0.63 (0.57, 0.69), <.001	0.22	1.56	—	0.64 (0.57, 0.70), <.001	0.27	1.68	—
BMI, kg/m^2^	0.52 (0.46, 0.58), .552	0.11	24.08	—	0.52 (0.46, 0.59), 0.464	0.12	24.35	—
ALM								
ALM, kg	0.57 (0.50, 0.63), .038	0.12	15.83	—	0.58 (0.51, 0.65), .009	0.20	22.62	—
ALM/weight, kg/kg	0.51 (0.45, 0.58), .663	0.11	0.22	—	0.52 (0.46, 0.59), 0.422	0.10	0.28	—
ALM/height, kg/m	0.54 (0.48, 0.60), .232	0.09	10.22	—	0.55 (0.49, 0.62), .073	0.16	12.29	—
ALM/BMI kg/kg/m^2^	0.59 (0.53, 0.65), .004	0.21	0.56	—	0.59 (0.52, 0.65), .005	0.23	0.80	—
ALM/height^2^ kg/m^2^	0.51 (0.45, 0.57), .841	0.08	7.24	—	0.51 (0.45, 0.58), 0.647	0.13	6.67	—
Grip strength (GR)								
GR, kg	0.68 (0.63, 0.74),<.001	0.28	20.50	<20.00^*^	0.64 (0.58, 0.70), <.001	0.27	31.05	<35.50 kg^*^
GR/weight, kg/kg	0.64 (0.58, 0.69), <.001	0.25	0.30	<0.34^*^	0.58 (0.51, 0.64), .01	0.19	0.38	<0.45^*^
GR/height, kg/m	0.67 (0.61, 0.73), <.001	0.29	13.59	13.13	0.63 (0.57, 0.70), <.001	0.25	18.41	<22.00
GR/height^2^ kg/kg/m^2^	0.65 (0.59, 0.71), <.001	0.27	8.40	<8.25	0.62 (0.56, 0.68), <.001	0.19	9.67	<12.13
GR/BMI, kg/kg/m^2^	0.67 (0.61, 0.72), <.001	0.30	0.71	<0.79^*^	0.61 (0.54, 0.67), <.001	0.28	1.09	<1.05^*^
GR/ALM, kg/kg	0.64 (0.58, 0.70), <.001	0.25	1.15	1.36	0.59 (0.53, 0.66), .002	0.26	1.48	1.60

*Notes*: ALM = appendicular lean mass; BMI = body mass index; CI = confidence interval; GR = grip strength; SDOC = Sarcopenia Definitions and Outcomes Consortium.

^*^Denotes putative sarcopenia variables identified in the SDOC primary analysis.

For slow walking speed (<0.8 m/s), CART models identified GR/height <14 kg/m and GR <22.5 kg as the primary nodes in women and men, respectively (Supplementary Figures 1A and1B, respectively). Women with GR/height <14 kg/m were over one and a half times more likely to have slow walking speed than those with GR/height ≥14 kg/m. Men with GR <22.5 kg were over 6 times more likely to have slow walking speed than those with GR ≥22.5 kg, however, only 28 men in the sample had GR <22.5 kg. In women, CART models generated a secondary node of age <81.5 years and a tertiary node of ALM/BMI ≥0.56 kg/m^2^. Women 81.5 years and older with GR/height ≥14 kg/m were twice as likely to have slow walking speed than those <82 years with GR/height ≥14 kg/m.

CART identified variable importance for slow walking speed in women and men (Supplementary Table 5). In women, all 6 GR variables were identified as the most important variables for predicting walking speed <0.8 m/s (importance range = 8%–13%), with ALM/BMI and ALM/weight being seventh and eighth most important, respectively, followed by age. In men, all 6 GR variables were identified as the most important variables for predicting slow walking speed <0.8 m/s (range = 11%–24%). No ALM variables (except as adjustments for GR) or age were identified as important.

The optimal cut points identified by the Youden Index for slow walking speed (<0.8 m/s) for GR, GR/weight, and GR/BMI for women were <20.50 kg, <0.30 kg/kg, and <0.71 kg/kg/m^2^, respectively; and for men were <31.05 kg, <0.38 kg, and <1.09 kg/kg/m^2^, respectively (Table 3).

**Table 3.** Proportionate Agreement and Cohen’s Kappa for SDOC Grip Strength Cut Points and Optimal Cut Points Determined by Youden Index (Table 2) in Cohort 3 (*Strength Cohort*)

	Agreement Cohen’s Kappa (95% CI), *p* Value; Proportionate Agreement (%)					
	Women *N* = 2 440			Men *N* = 2 045		
Handgrip strength cut points	ANZ Grip Strength, kg <20.50	ANZ Grip Strength/Weight, kg/kg <0.30	ANZ Grip Strength/BMI kg/kg/m^2^ <0.71	ANZ Grip Strength, kg <31.05	ANZ Grip Strength/Weight, kg/kg <0.38	ANZ Grip Strength/BMI kg/kg/m^2^ <1.09
SDOC grip strength, kg Women <20 Men <35.5	**0.94 (0.92, 0.95), <.001; 97.0%**	0.74 (0.71, 0.76), <.001; 87.1%	0.80 (0.78, 0.82), <.001; 90.5%	**0.84 (0.82, 0.87), <.001; 92.4%**	0.78 (0.75, 0.80), <.001; 89.1%	0.79 (0.76, 0.82), <.001; 90.0%
SDOC grip strength/weight, kg/kg Women <0.34 Men <0.45	0.63 (0.60, 0.66), <.001; 81.2%	**0.80 (0.78, 0.82), <.001; 90.0%**	0.71 (0.68, 0.74), <.001; 85.3%	0.66 (0.63, 0.70), <.001; 83.1%	**0.74 (0.71, 0.76), <.001; 86.8%**	0.71 (0.68, 0.74), <.001; 85.3%
SDOC grip strength/BMI kg/kg/m^2^ Women <0.79 Men <1.05	0.74 (0.71, 0.77), <.001; 87.1%	0.89 (0.87, 0.91), <.001; 94.6%	**0.86 (0.84, 0.88), <.001; 93.2%**	0.86 (0.83, 0.88), <.001; 93.5%	0.92 (0.90, 0.94), <.001; 96.4%	**0.97 (0.96, 0.98), <.001; 98.6%**

*Notes*: ANZ = Australia and New Zealand; CI = confidence interval; SDOC = Sarcopenia Definitions and Outcomes Consortium.

Figures in bold reflect the comparison of equivalent measures between ANZ and SDOC.

Table 3 presents the agreement by CK between Cohort 3 (*strength cohort*) (*n* = 2 440 women, *n* = 2 045 men) ROC and Youden Index-derived cut points for GR (and adjustments) predicting slow walking speed and the SDOC-generated cut points. Comparing equivalent measures, all variables showed “almost perfect agreement” between cut points (range 0.80–0.97) in both women and men (*p* value <.001), except GR/weight in men, which showed “substantial” agreement of 0.74 (*p* value <.001). Proportionate agreement (%) was high across all equivalent measures (range 86.8–97.0).

Table 4 presents the prevalence estimates of probable sarcopenia (either low GR or slow walking speed) and sarcopenia for Cohorts 1 to 4 by SDOC and EWGSOP2 cut points and definitions and respective agreement by CK and proportionate agreement. In women, the highest prevalence of probable sarcopenia (63.2%) was found in Cohort 1 (*performance and strength cohort*) by EWGSOP2 (walking speed ≤0.8 m/s), and the lowest prevalence in Cohort 4 (*TUG cohort*) by EWGSOP2 (TUG test ≥20 s) with no women (0.0%) exceeding this cut point. The highest prevalence of sarcopenia in women (37.2%) was found using the SDOC definition in Cohort 1 (*performance and strength cohort*), and the lowest prevalence of sarcopenia (1.5%) was found using the EWGSOP2 definition in Cohort 3 (*strength cohort*). There was no definition agreement using CK (95% CI: *p* value) and low agreement using proportionate agreement (%) in Cohort 1 for sarcopenia in women (0.07 [0.03, 0.12; .008]; 34.2%, and 0.14 [0.07, 0.20; <.001]; 40.1%, when using EWGSOP2, ALM/height^2^, and ALM cut points, respectively).

**Table 4.** Sex-Stratified Comparison Between SDOC and EWGSOP2 Definitions of Probable Sarcopenia (Low Strength or Physical Performance) and Sarcopenia in Four Australian and New Zealand Cohorts by Prevalence and Agreement by Cohen’s Kappa

Cohort	SDOC			EWGSOP2						
	Low Strength or Physical Performance		Sarcopenia	Low Strength or Physical Performance			Appendicular Lean Mass		Sarcopenia	
	Low GR *W* <20 kg *M* <35.5 kg	Walking Speed <0.8 m/s	Low GR and Slow Walking Speed	Low GR *W* <16 kg *M* <27 kg	Walking Speed ≤0.8 m/s	TUG ≥20 s	ALM *W* <15 kg *M* <20 kg	ALM/ht^2^ *W* <5.5 kg/m^2^ *M* <7 kg/m^2^	Low GR and ALM	Low GR and ALM/ht^2^
Cohort 1—Performance and strength cohort										
Women	295/1 121 (26.3)	228/364 (62.6)	135/363 (37.2)	132/1 121 (11.8)	230/364 (63.2)	122/1 111 (11.0)	230/1 151 (20.0)	76/1 148 (6.6)	60/1 111 (5.4)	24/1 108 (2.2)
Men	260/682 (38.1)	110/675 (16.3)	61/674 (9.1)	52/582 (7.6)	112/675 (16.6)	46/678 (6.8)	54/680 (7.9)	61/680 (9.0)	18/679 (2.7)	16/679 (2.4)
Cohort 2—performance cohort										
Women	—	314/995 (31.6)	—	—	315/995 (31.7)	8/984 (0.8%)	104/312 (33.3)	52/312 (16.7)	—	—
Men	—	149/461 (32.3)	—	—	149/461 (32.3)	0/453 (0.0%)	16/172 (9.3)	17/172 (9.9)	—	—
Cohort 3—strength cohort										
Women	887/2 440 (36.4)	—	—	704/2 440 (28.9)	—	1/169 (0.6)	208/854 (24.4)	66/852 (7.7)	34/840 (4.0)	13/840 (1.5)
Men	881/2 045 (43.1)	—	—	608/2 045 (29.7)	—	—	55/737 (7.5)	42/737 (5.7)	13/729 (1.8)	7/729 (1.0)
Cohort 4—Timed Up and Go cohort										
Women	—	—	—	—	—	0/49 (0.0)	52/134 (38.8)	21/134 (15.7)	—	—
Men	—	—	—	—	—	0/16 (0.0)	9/49 (18.4)	6/49 (12.2)	—	—

*Notes*: ALM = appendicular lean mass; EWGSOP2 = revised European Working Group for Sarcopenia in Older People; M = men; SDOC = Sarcopenia Definitions and Outcomes Consortium; W = women.

Cohen’s Kappa (95% CI; *p* value), proportionate agreement: Cohort 1 (women), SDOC vs EWGSOP2 (ALM) = 0.14 (0.07, 0.20; <.001), 40.1%; SDOC vs EWGSOP2 (ALM/ht^2^) = 0.07 (0.03, 0.12; .008), 34.2%; Cohort 1 (men), SDOC vs EWGSOP2 (ALM) = 0.21 (0.15, 0.25; <.001), 61.3%; SDOC vs EWGSOP2 (ALM/ht^2^) = 0.17 (0.12, 0.22; <.001), 59.9%; Cohort 2 (women), SDOC slow walking speed vs EWGSOP2 slow walking speed = 0.99 (0.99, 1.00; <.001), 99.9%; Cohort 2 (men), SDOC slow walking speed vs EWGSOP2 slow walking speed = 0.99 (0.97, 1.00; <.001), 99.3%; Cohort 3 (women), SDOC low GR vs EWGSOP2 (ALM) = 0.23 (0.19, 0.27; <.001), 64.9%; SDOC low GR vs EWGSOP2 (ALM/ht^2^) = 0.27 (0.23, 0.30; <.001), 73.9%; Cohort 3 (men), SDOC low GR vs EWGSOP2 (ALM) = 0.22 (0.18, 0.26; <.001), 69.7%; SDOC low GR vs EWGSOP2 (ALM/ht^2^) = 0.17 (0.14, 0.21; <.001), 68.0%.

In men, the highest prevalence of probable sarcopenia (43.1%) was found in Cohort 3 (*strength cohort*) by SDOC (GR <35.5 kg), and the lowest prevalence in Cohorts 2 (*performance cohort*) and 4 (*TUG cohort*) by EWGSOP2 (TUG ≥20 s) with no men exceeding this cut point in either cohort (Table 4). The highest prevalence of sarcopenia in men (9.1%) was found using the SDOC definition in Cohort 1 (*performance and strength cohort*), and the lowest prevalence of sarcopenia (1.0%) using the EWGSOP2 definition in Cohort 3 (*strength cohort*). There was no definition agreement using CK (95% CI; *p* value) and low agreement using proportionate agreement (%) in Cohort 1 for sarcopenia in men (0.17 [0.12, 0.22; <.001]; 59.9%, and 0.21 [0.15, 0.25; <.001]; 61.3%, when using EWGSOP2, ALM/height^2^, and ALM cut points, respectively).

## Discussion

This study replicated the methodology of the SDOC in an ANZ pooled cohort by showing that GR (with and without body size and composition adjustments) was consistently the strongest discriminator of slow walking speed (<0.8 m/s) in women and men. We also replicated the SDOC finding that DXA-derived ALM was not a useful discriminator of slow walking speed. Second, we found that the GR cut points derived in this study that best predict slow walking speed in ANZ women and men showed near-perfect agreement with the cut points generated in the SDOC analysis of older adults mostly from the United States (2). Finally, we showed that there was no definition agreement between SDOC and EWGSOP2 cut points for probable and confirmed sarcopenia in women and men, and the estimated prevalence of sarcopenia varies significantly within and between ANZ adult populations depending on the definition applied.

This study is presented at a time of great change in the sarcopenia field. The search for a global definition of sarcopenia is ongoing and remains elusive. Recently, the Global Initiative in Sarcopenia (GLIS) announced a forthcoming worldwide Delphi process whose goal is to produce an inclusive definition of sarcopenia (37). In ANZ, the ANZSSFR Task Force recently published clinical guidelines in which 67 topic experts achieved consensus through a modified Delphi process to, among other recommendations, adopt the EWGSOP2 sarcopenia definition (5,13). The process also called for definition validation in the region (13). This novel replication of the SDOC findings and prevalence estimates using both SDOC and EWGSOP2 sarcopenia definitions in large ANZ cohorts will represent the region and may add to the GLIS process. Our work highlights the limitations of, and low sarcopenia prevalence estimated by, the EWGSOP2 definition in ANZ community-dwelling older adults. Identification of older adults with sarcopenia (which is more likely by applying the SDOC definition) is critical to support person-centered management aimed at mitigating adverse outcomes of sarcopenia. Clinicians may consider our findings when applying the EWGSOP2 sarcopenia definition to older adults in ANZ.

The analytic approach in our study replicates key methodological elements of the SDOC approach but differs by inclusion criteria (eg, age), cohort size, and breadth of variables examined (2,17,18,38). In concert with the SDOC, we selected walking speed <0.8 m/s as the primary outcome for numerous reasons. First, walking speed has been recognized as the “6th vital sign” (39) and is an established predictor of adverse outcomes across adulthood (18,34). Second, in recognition of its clinical significance, walking speed <0.8 m/s has been consistently used as the cut point in recent sarcopenia definitions (40). Third, as compared with GR, walking speed improves with intervention (41), and an increase of 0.1 m/s has been recognized as clinically meaningful change (42). Finally, clinically meaningful change in walking speed has positive functional benefits for individuals (43) and reduces health costs (44). We did not perform predictive models for walking speed <0.6 m/s, <1.0 m/s, or as a continuous variable given the SDOC resolution was to accept walking speed <0.8 m/s as the preferred outcome and cut point.

Critically, our study differed from SDOC in that we included adults of all ages and provided age medians and ranges, as opposed to including only adults 65 and older. We broadened the age inclusion criteria for multiple reasons. First, muscle strength declines correlate with normal walking speed across adulthood and are not limited to older adults (45,46). In other words, adults younger than 65 years with muscle weakness are also more likely to have slow walking speed, thus should be equally afforded the opportunity for investigation and intervention as older adults. Second, the ANZSSFR Task Force recommendation three (13) states, “…adults at risk of sarcopenia should be assessed annually or after the occurrence of a major health event.” In this context, “at risk” includes persons with comorbidities likely to increase the risk of sarcopenia (eg, cardiac failure) and those whose ethnicity carries heightened epigenetic and sociocultural risks for multimorbidity such as Māori, Aboriginal, and Torres Strait Islander persons (47); priority populations in our region (48). Given we did not have all ethnicity information available, and CART modelling does not account for comorbidities, the age of inclusion was broadened to ensure persons less than 65 years but with heightened risk for sarcopenia were represented. This decision was validated by the SDOC findings, which showed that the inclusion of Black race and Chinese variables in the models did not alter variable importance or selection (2), despite established evidence that normative values of GR and walking speed vary across racial groups (10,49).

The cohort used for CART analyses was significantly smaller in our study compared with the SDOC. These sample size discrepancies did not result in different calculated cut points for GR and adjustments using sensitivity analyses and Youden Indices; CK showed the agreement to be substantial or near-perfect. However, our CART-generated tree in men showed a primary node of GR <22.5 kg, much lower than the <35.5 kg shown in SDOC and found in the ROC analyses. This could be explained by an overfitted tree, whereby only 28 men (4.1% of sample) had GR <22.5 kg, of which 24 (85.7%) had walking speed <0.8 m/s. Based on this finding, we applied the Youden Index cut points for comparison to SDOC cut points, which better reflect the discriminating capacity of each variable given the sample size.

The SDOC used 35 candidate variables, including DXA measures for fat mass (as adjustments/denominators), arm and leg lean mass, and adjustments of primary variables for BSA. These additional variables were not included in our study; however, it was noted that none was deemed to have high variable importance or meaningfully contribute to the predictive model in the SDOC analysis (2). Of note, chair sit-to-stand test (STS), a measure of proximal muscle strength and considered by some a physical performance measure (50,51), was not included as a candidate variable in the SDOC or our analysis owing to data availability (2). However, recent CART models have demonstrated that STS is a superior predictor of incident mobility disability in older men (36). Including STS in future predictive models may enhance our understanding of the comparative discriminating capacity of STS compared with GR for slow walking speed or other adverse outcomes of interest.

Measures of ALM by DXA were found to be unimportant variables for predicting slow walking speed and were not selected as primary or secondary nodes in women or men. This finding differed slightly from the SDOC analysis in which ALM/appendicular fat mass (ALM/AFM) was a secondary node in women, albeit representing a small subsample and thus not expected to yield significant practical benefit (2). However, our study did not include measures of fat mass, which would explain this difference. The finding that ALM is not an important predictor of slow walking speed does not diminish the importance of low muscle mass in predicting adverse outcomes in older people. ALM and muscle mass are not equivalent; ALM is a surrogate of and overestimates muscle mass (3). In recent studies of accurate measures of muscle mass (such as the D_3_-creatine dilution method), muscle mass is strongly associated with negative outcomes in older adults (36,52,53) in contrast to studies showing mixed associations of ALM with similar outcomes (53–55). The EWGSOP2 sarcopenia definition requires the presence of low muscle quality or quantity (eg, by DXA ALM) to confirm sarcopenia. Our study showed a low prevalence of sarcopenia in women and men when including DXA ALM measures and no definition agreement with SDOC, which adds to the body of evidence suggesting that these definitions may measure different characteristics (18). The finding in our Cohort 3 (*strength cohort*) analysis showing no agreement between SDOC low GR (probable sarcopenia) and EWGSOP2 low GR and low ALM or ALM/ht^2^ (sarcopenia) suggests that ALM may be the variable driving the poor agreement between definitions, beyond that which would be expected by different GR cut points.

The high variability of sarcopenia prevalence between cohorts and definitions found in our study reflects the variability and poor definition agreement in recent studies of ANZ older adults (20,22,25,26,56–58). In the GOS, population-specific cut points of low GR, ALM, TUG (men), and walking speed (women) were calculated as 2 standard deviations (*SD*s) below the mean in adults aged 60–96 years. Sarcopenia prevalence ranged from 0.9% to 10.4% in women, and 1.6% to 18.4% in men, depending on the variables selected to define sarcopenia (20). In the same cohort applying the EWGSOP2 definition of sarcopenia, the prevalence of sarcopenia was found to be lower; 2.3% in women and 0.5% in men (22). In a study of community-dwelling women with average age of 80 years in Perth, Australia, sarcopenia prevalence varied from 9.4% to 24.1% depending on the definition applied and showed mixed associations with mortality (56). The application of EWGSOP1 to 1 486 community-dwelling older men in Sydney, Australia, found sarcopenia prevalence of 15.9% (58). In a New Zealand cohort of older women and men residing in long-term care (average age 86 years), sarcopenia prevalence was estimated at 41.0% using the EWGSOP1 definition. The sarcopenia prevalence estimates from our study fall within the range of these studies in the region (women, 1.5%–37.2%; men, 1.0%–9.1%), excepting one study which applied EWGSOP1 (58). However, the wide range of sarcopenia prevalence and low or absent agreement between definitions highlights both the challenges in interpreting and generalizing these findings and the need for global consensus.

This study was strengthened by replication of the established and recognized SDOC methodology and by adherence to the TRIPOD guidelines (2,27). The CART methodology is agnostic and thus included variables were considered equally by the model. For the first time in the region, this study also combined large ANZ cohorts to establish representative cut points for low GR. A strength of this study also exists in its replication of the SDOC findings, rather than the generation of another definition of sarcopenia. Our study was limited by the small number of participants in Cohort 1 (*performance and strength cohort*) on whom CART was performed. We also did not have ethnicity and race data, which may have affected generalizability. Future studies should specifically include priority populations in our region, particularly Aboriginal, Torres Strait, and Māori adults. Other limitations reflect those described by the SDOC; slow walking speed was the only outcome evaluated, and practicality and volitional effort of GR are not considered (2). Finally, future cohort studies should evaluate associations of adverse outcomes with sarcopenia defined by the cut points validated in ANZ.

In conclusion, this study comprising a large cohort of ANZ women and men replicated findings of the SDOC analysis: GR with and without adjustments is the primary discriminating variable for slow walking speed. Lean mass variables were not important predictors of slow walking speed (except as adjustments). When ALM variables were included in the definition of sarcopenia (EWGSOP2), sarcopenia prevalence estimates were lower in both women and men than when lean mass variables were excluded. Further, the SDOC and EWGSOP2 definitions showed no agreement in this pooled cohort study, suggesting that these definitions measure different characteristics and are likely to predict sarcopenia-related outcomes differently. Our study suggests that DXA-derived approximations may have limited value in a sarcopenia definition if the key outcome is slow walking speed. Although the ANZSSFR consensus guidelines recommended the use of the EWGSOP2, our analysis shows that prevalence estimates significantly differ when using EWGSOP2 and SDOC definitions in an ANZ population. Overall, this work supports the inclusion of GR measures in the definition of current and future sarcopenia definitions.

## Supplementary Material

glad165_suppl_Supplementary_Material

## Data Availability

These data are protected by Data Transfer Agreement. Readers may contact individual data custodians of the respective cohorts for queries regarding data access.

## Statistical Analysis

J.Z. performed the statistical analyses and is independent of any commercial funder. He had full access to all the data in the study and takes responsibility for the integrity of the data and the accuracy of the data analyses.
