# Supplementary material for: Predicting Slow Walking Speed From a Pooled Cohort Analysis: Sarcopenia Definitions, Agreement, and Prevalence in Australia and New Zealand
Source: J Gerontol A Biol Sci Med Sci. 2023 Jul 10;78(12):2415–25. doi: 10.1093/gerona/glad165 (PMC10692428; doi:10.1093/gerona/glad165)
Supplement: glad165_suppl_Supplementary_Material [file glad165_suppl_supplementary_material.docx]

**Supplementary Material**

Supplemental eTable S1 – Revised European Working Group on Sarcopenia in Older People and Sarcopenia Definition and Outcomes Consortium definitions

Supplemental eTable S2 – Cohort descriptions, grip strength and walking speed protocols for included cohorts

Supplemental eTable S3 – Candidate variables competing for slow walking speed (<0.8 m/s) in CART analysis

Supplemental eTable S4 – Characteristics of women and men with and without slowness (Cohort 2) and with and without low grip strength (Cohort 3 – *performance cohort*)

Supplemental eTable S5 – Cohort 1 CART analysis-derived variable importance for predicting walking speed <0.8 m/s in women and men

Supplementary Figure S1A – Classification and regression tree for discriminating slow walking speed (<0.8 m/s) in women

Supplementary material reference list

**Supplemental eTable S1 – Revised European Working Group on Sarcopenia in Older People and Sarcopenia Definition and Outcomes Consortium definitions**

| **Component** | **Cut points** |
| --- | --- |
| **Revised European Working Group on Sarcopenia in Older People** (1)  Probable sarcopenia = Low muscle strength  Sarcopenia = Low muscle strength AND low muscle quality or quantity  Severe sarcopenia = Low muscle strength AND low muscle quality or quantity AND low physical performance | |
| Low muscle quantity  Low muscle strength  Low physical performance | ASM adjusted for height (m^2^) using whole-body DXA: Men: <7.0 kg/m^2^ Women: <5.5 kg/m^2^ ASM using DXA: Men: <20.0 kg  Women: <15.0 kg  Hand grip strength using dynamometer:  Men: <27 kg  Women: <16 kg  Chair stand:  >15 s for 5 rises  Walking speed: ≤0.8m/s  SPPB: <8 point score  TUG: >12 s*  400 m walk test: non-completion of >6 min |
| **Sarcopenia Definition and Outcomes Consortium** (2)  Sarcopenia = Low muscle strength (grip strength) AND low physical performance (walking speed) | |
| Low muscle strength  Low physical performance | Unadjusted hand grip strength using dynamometer:  Men: <35.5 kg  Women: <20 kg  Adjusted hand grip strength:  *Grip strength/BMI*  Men: <1.05 kg/kg/m^2^  Women: <0.79 kg/kg/m^2^  *Grip strength/TBF*  Men: <1.66 kg/kg  Women: <0.65kg/kg  *Grip strength/arm lean mass*  Men: <6.08kg/kg  Women: <3.26 kg/kg  *Grip strength/weight*  Men: <0.45 kg/kg  Women: <0.337 kg/kg  Walking speed: <0.8 m/s |

ASM = Appendicular skeletal muscle mass. BIA = Bioelectrical impedance analysis. DXA = Dual energy x-ray absorptiometry. SPPB = Short physical performance battery. TUG = Timed Up and Go test. TBF = total body fat.

**Supplemental eTable S2 – Cohort descriptions, grip strength and walking speed protocols for included cohorts**

|  |  |  | **Grip strength** | | | | | **Walking speed** | | |
| --- | --- | --- | --- | --- | --- | --- | --- | --- | --- | --- |
| Study | Eligibility | Year | Equipment | Hand-dominance | Position | Trials | Notes | Distance | Trials | Exclusions |
| Australian Institute for Musculoskeletal Science Falls and Facture Clinic (FFC) (3) | Cross-sectional study of community-dwelling adults >65 years, referred to a falls and fracture clinic in Melbourne, Australia | Baseline | Handheld Jamar hydraulic dynamometer | NA | Seated upright in an armless chair with elbow flexed at 90° | 3 trials of each hand | Best performance on either side used in analysis | 4m using the GAIT Rite system | 3 trials with best used for analysis | Exclusion from the performance tests will be based on an individualized assessment of impairments and safety concerns. |
| Geelong Osteoporosis Study (GOS) (4) | Prospective cohort of community-dwelling adults selected at baseline as age-stratified samples from electoral roll in Victoria, Australia | 15 | Handheld Jamar hydraulic dynamometer (women) and electronic handheld dynamometer for men, adjusted using transformation equation (4) | NA | Seated upright in an armless chair with elbow flexed at 90° | 2-3 trial on each hand | Best performance on either side used in analysis | 4m using trained personnel | 1 trial | Exclusion from the performance tests will be based on an individualized assessment of impairments and safety concerns. |
| Dubbo Osteoporosis Epidemiology Study (DOES) (5) | Prospective cohort of community-dwelling adults aged >60 years in Dubbo, New South Wales, Australia | Baseline | NA | NA | NA | NA | NA | 6m using trained personnel | 1 trial | Exclusion from the performance tests will be based on an individualized assessment of impairments and safety concerns. |
| Vital D study (Vital D) (6) | Double blind, randomized, placebo controlled vitamin D intervention study in women >70 years in Southern Victoria, Australia. | Baseline | NA | NA | NA | NA | NA | 4m using the GAIT Rite system, with 3m ‘lead in’ and 2m ‘lead off’ distance | 3 trials, data from trials 2 and 3 combined to provide mean value | Exclusion from the performance tests will be based on an individualized assessment of impairments and safety concerns. |
| North West Adelaide Health Study (NWAHS) (7) | Prospective cohort of community-dwelling, randomly selected aged 18 and over at the time of recruitment (1999-2003) from the Northern and Western suburbs of Adelaide, South Australia, Australia | Stage 3, 8 – 10 year visit | Handheld Jamar hydraulic dynamometer | NA | Seated upright in an armless chair with elbow flexed at 90° | 3 trials of each hand | Best performance on either side used in analysis | NA | NA | NA |
| Tasmanian Older Adult Cohort (TASOAC) (8) | Prospective cohort of community-dwelling, randomly selected men and women aged 50-79 years from Southern Tasmania | Baseline | North Coast bulb dynamometer | NA | Seated upright in an armless chair with elbow flexed at 90° | 2 trials of each hand | Best performance on either side used in analysis | NA | NA | NA |
| Women’s Healthy Ageing Project (WHAP) (9) | Prospective cohort of community-dwelling women aged 50 years and older, with sample at year 26 all age >70 years | 22 | Handheld Jamar hydraulic dynamometer | NA | Seated upright in an armless chair with elbow flexed at 90° | 3 trials of each hand | Best performance on either side used in analysis | NA | NA | NA |
| Age Concern Otago (OTAGO) (10) | Prospective cohort of community-dwelling adults aged 65-94 years in Otago, New Zealand | Baseline | NA | NA | NA | NA | NA | NA | NA | NA |

**Supplemental eTable S3 – Candidate variables competing for slow walking speed (<0.8 m/s) in CART analysis**

| No. | Variable | Abbreviation |
| --- | --- | --- |
| 1 | Age (years) | AGE |
| 2 | Weight (kg) | WT |
| 3 | Height (m) | HT |
| 4 | Body Mass Index (kg/m^2^) | BMI |
| 5 | Hand grip strength (kg) | GR |
| 6 | Hand grip strength (kg) / Height (m) | GRHGT |
| 7 | Hand grip strength (kg) / Weight (kg) | GRWGT |
| 8 | Hand grip strength (kg) / Height^2^ (m^2^) | GRHTSQ |
| 9 | Hand grip strength (kg) / Body Mass Index (kg/m^2^) | GRBMI |
| 10 | Appendicular Lean Mass (kg) | GRALM |
| 11 | Appendicular Lean Mass (kg) | ALM |
| 12 | Appendicular Lean Mass (kg) / Height (m) | ALMHGT |
| 13 | Appendicular Lean Mass (kg) / Weight (kg) | ALMTBW |
| 14 | Appendicular Lean Mass (kg) / Height^2^ (m^2^) | ALMHSQ |
| 15 | Appendicular Lean Mass (kg) / Body Mass Index (kg/m^2^) | ALMBMI |

**Supplemental eTable S4 – Characteristics of women and men with and without slowness (Cohort 2) and with and without low grip strength (Cohort 3 – *performance cohort*)**

|  | **Women** | | | | | | **Men** | | | | | |
| --- | --- | --- | --- | --- | --- | --- | --- | --- | --- | --- | --- | --- |
|  | Walking speed (Cohort 2, N = 995) | | | Grip strength (Cohort 3, N = 2440) | | | Walking speed (Cohort 2, N = 461) | | | Grip strength (Cohort 3, N = 2045 | | |
| **Characteristic** | <0.8 m/s  (N = 314) | >0.8 m/s  (N = 681) | P value | <20 kg  (N = 887) | >20 kg  (N = 1553) | P value | <0.8 m/s  (N = 146) | >0.8 m/s  (N = 315) | P value | <35.5 kg  (N = 881) | >35.5 kg  (N = 1164) | P value |
| Age (yrs) | 70.56 | 69.33 | <.001 | 65.31 | 54.65 | <.001 | 70.57 | 68.78 | <.001 | 65.30 | 51.28 | <.001 |
| Height (m) | 1.58 | 1.60 | <.001 | 1.59 | 1.62 | <.001 | 1.72 | 1.74 | .005 | 1.72 | 1.76 | <.001 |
| Total body weight (kg) | 74.10 | 70.01 | <.001 | 71.40 | 73.52 | .002 | 86.30 | 86.02 | .838 | 83.05 | 88.09 | <.001 |
| BMI (kg/m^2^) | 29.5 | 27.2 | <.001 | 28.2 | 28.1 | .723 | 29.0 | 28.3 | .096 | 27.9 | 28.3 | .072 |
| Walking speed (m/s) | 0.68 | 1.02 | <.001 | - | - | - | 0.70 | 0.95 | <.001 | - | - | - |
| Grip strength (kg) | - | - | - | 12.24 | 28.27 | <.001 | - | - | - | 20.54 | 47.66 | <.001 |
| Grip strength (kg) / weight (kg) | - | - | - | 0.18 | 0.40 | <.001 | - | - | - | 0.26 | 0.55 | <.001 |
| Grip strength (kg) / BMI (kg/m^2^) | - | - | - | 0.45 | 1.05 | <.001 | - | - | - | 0.75 | 1.72 | <.001 |
| Appendicular lean mass (kg) | 15.44 | 16.11 | .019 | 15.84 | 17.03 | <.001 | 22.52 | 24.65 | <.001 | 22.71 | 26.30 | <.001 |
| Appendicular lean mass (kg) / height^2^ (m^2^) | 6.13 | 6.22 | .344 | 6.41 | 6.57 | .018 | 7.82 | 8.20 | .007 | 7.96 | 8.59 | <.001 |

BMI = Body Mass Index.

**Supplemental eTable S5 – Cohort 1 CART analysis-derived variable importance for predicting walking speed <0.8 m/s in women and men**

| Variable rank | Women | | Men | |
| --- | --- | --- | --- | --- |
|  | Variable | Importance (%) | Variable | Importance (%) |
| 1 | Grip strength / height (kg/m) | 13 | Grip strength (kg) | 24 |
| 2 | Grip strength (kg) | 13 | Grip strength / height (kg/m) | 22 |
| 3 | Grip strength / height^2^ (kg/m^2^) | 12 | Grip strength / height^2^ (kg/m^2^) | 18 |
| 4 | Grip strength / BMI (kg/kg/m^2^) | 10 | Grip strength / BMI (kg/kg/m^2^) | 14 |
| 5 | Grip strength / weight (kg/kg) | 8 | Grip strength / ALM (kg/kg) | 11 |
| 6 | Grip strength / ALM (kg/kg) | 8 | Grip strength / weight (kg/kg) | 11 |
| 7 | ALM/ BMI (kg/kg/m^2^) | 8 |  |  |
| 8 | ALM / weight (kg/kg) | 7 |  |  |
| 9 | Age (years) | 7 |  |  |
| 10 | Height (m) | 5 |  |  |

BMI = Body mass index; ALM = Appendicular lean mass

**Supplementary Figure S1A – Classification and regression tree for discriminating slow walking speed (<0.8 m/s) in women**


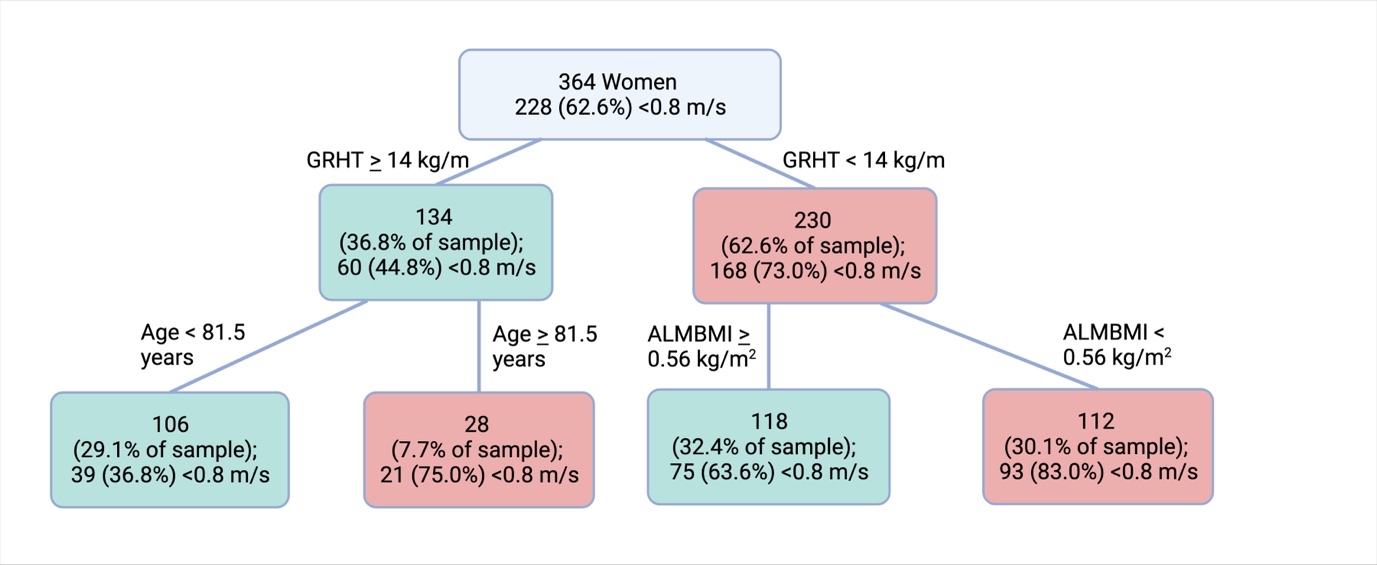


GRHT = Grip strength (kg) / height (m). ALMBMI = Appendicular lean mass (kg) / body mass index (kg/m^2^).

**Supplementary Figure S1B – Classification and regression tree for discriminating slow walking speed (<0.8 m/s) in men.**


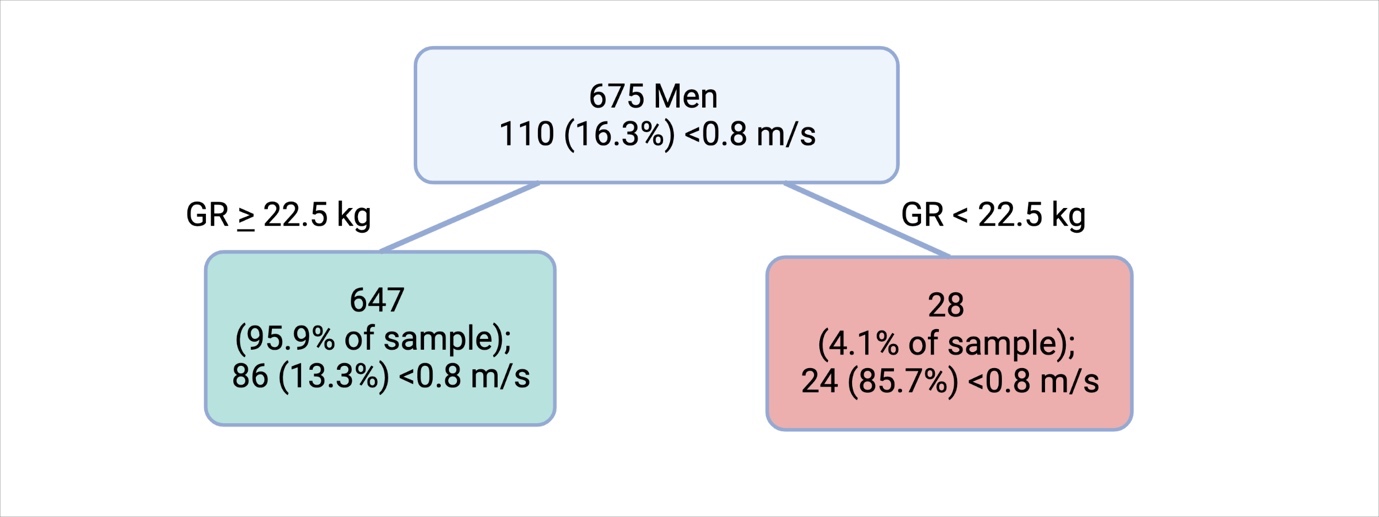


GRHT = Grip strength (kg) / height (m). ALMBMI = Appendicular lean mass (kg) / body mass index (kg/h^2^). GR = Grip strength (kg)

**Supplementary material references**

1. Cruz-Jentoft AJ, Bahat G, Bauer J, et al. Sarcopenia: revised European consensus on definition and diagnosis. *Age Ageing*. 2019;48(1):16-31. doi:10.1093/ageing/afy169.

2. Manini TM, Patel SM, Newman AB, et al. Identification of Sarcopenia Components That Discriminate Slow Walking Speed: A Pooled Data Analysis. *J Am Geriatr Soc*. 2020;68(7):1419-1428. doi:10.1111/jgs.16524.

3. Kirk B, Zanker J, Bani Hassan E, Bird S, Brennan-Olsen S, Duque G. Sarcopenia Definitions and Outcomes Consortium (SDOC) Criteria are Strongly Associated With Malnutrition, Depression, Falls, and Fractures in High-Risk Older Persons. *J Am Med Dir Assoc*. 2021;22(4):741-745. doi:10.1016/j.jamda.2020.06.050.

4. Sui SX, Holloway-Kew KL, Hyde NK, et al. Prevalence of Sarcopenia Employing Population-Specific Cut-Points: Cross-Sectional Data from the Geelong Osteoporosis Study, Australia. *J Clin Med*. 2021;10(2):343. doi:10.3390/jcm10020343.

5. Bliuc D, Tran T, Alarkawi D, Nguyen T v., Eisman JA, Center JR. Secular Changes in Postfracture Outcomes Over 2 Decades in Australia: A Time-Trend Comparison of Excess Postfracture Mortality in Two Birth Controls Over Two Decades. *J Clin Endocrinol Metab*. 2016;101(6):2475-2483. doi:10.1210/jc.2016-1514.

6. Sanders KM, Stuart AL, Merriman EN, et al. Trials and tribulations of recruiting 2,000 older women onto a clinical trial investigating falls and fractures: Vital D study. *BMC Med Res Methodol*. 2009;9(1):78. doi:10.1186/1471-2288-9-78.

7. Grant JF, Taylor AW, Ruffin RE, et al. Cohort Profile: The North West Adelaide Health Study (NWAHS). *Int J Epidemiol*. 2009;38(6):1479-1486. doi:10.1093/ije/dyn262.

8. Zhai G, Cicuttini F, Srikanth V, Cooley H, Ding C, Jones G. Factors associated with hip cartilage volume measured by magnetic resonance imaging: The Tasmanian Older Adult Cohort Study. *Arthritis Rheum*. 2005;52(4):1069-1076. doi:10.1002/art.20964.

9. Szoeke C, Coulson M, Campbell S, Dennerstein L. Cohort profile: Women’s Healthy Ageing Project (WHAP) - a longitudinal prospective study of Australian women since 1990. *Womens Midlife Health*. 2016;2(1):5. doi:10.1186/s40695-016-0018-y.

10. Waters DL, Hale LA, Robertson L, Hale BA, Herbison P. Evaluation of a Peer-Led Falls Prevention Program for Older Adults. *Arch Phys Med Rehabil*. 2011;92(10):1581-1586. doi:10.1016/j.apmr.2011.05.014.
